# Complete Genome Sequences of Two Salmonella enterica Strains Isolated from Chicken Carcass Rinse Water in Thailand

**DOI:** 10.1128/mra.01235-22

**Published:** 2023-01-04

**Authors:** Sudarat Ledlod, Phatcharaporn Thuamkaew, Phanthipha Luecha, Tantip Arigul, Worarat Kruasuwan, Natnicha Wankaew, Adison Khongchatee, Piroon Jenjaroenpun, Thidathip Wongsurawat

**Affiliations:** a Research Department, CPF Food Research and Development Center Co., Ltd., Lamsai, Phranakhon Si-Ayutthaya, Thailand; b Bacteriology Department, Animal Health Diagnostic Center, Bangkok, Thailand; c Division of Medical Bioinformatics, Research Department, Faculty of Medicine Siriraj Hospital, Mahidol University, Bangkok, Thailand; d Siriraj Long-read Lab, Faculty of Medicine Siriraj Hospital, Mahidol University, Bangkok, Thailand; University of Rochester School of Medicine and Dentistry

## Abstract

We report the circularized complete genome sequences, containing a circular chromosome and two circular plasmids, of strains SalSpp05 (4.9 Mbp) and SalSpp10 (4.8 Mbp), which were isolated from chicken carcass rinse water samples; the sequences were obtained by combining Oxford Nanopore Technologies long-read data and Illumina short-read data. Whole-genome alignments indicated that both strains belong to Salmonella enterica.

## ANNOUNCEMENT

Salmonella spp. are important foodborne pathogens that are distributed worldwide and are associated with poultry infections (1). Recently, the distribution of antimicrobial resistance (AMR) in Salmonella strains obtained from chickens could cause disease in humans in Thailand with the risk of unsuccessful treatment (2). Contamination with Salmonella impacts the process from farm to fork in the food industry and is usually detected by either processing swab samples or obtaining several sample types, such as samples from feed, equipment, fomite, diseased chicks, and carcasses (3). Since Salmonella enterica serovar Enteritidis has predominantly infected poultry hosts and evolved through adaptation to different host environments, routine Salmonella monitoring programs on poultry farms would improve food safety.

Here, carcass rinse water samples collected from Klangkoi chicken farm in Thailand were screened for Salmonella as part of routine surveillance, by growth in buffered peptone water medium at 37°C for 24 h. Genomic DNA (gDNA) was extracted by using ZymoBIOMICS DNA kits (Zymo Research, USA), and the quality of DNA was assessed using a NanoDrop spectrophotometer (Thermo Fisher Scientific, USA). The same gDNA was used for Illumina and Oxford Nanopore Technologies (ONT) sequencing. For short-read sequencing, a 100-bp paired-end library was constructed using the TruSeq Nano DNA kit and sequenced using an Illumina HiSeq 2500 system with the HiSeq Rapid SBS kit v2 (Illumina, USA). Long-read sequencing was performed using a rapid barcoding kit (SQK-RBK004; ONT, UK) and an R9.4.1 flow cell (FLO-MIN106) on a Nanopore MinION Mk1c system (ONT) in super accuracy mode, and data were demultiplexed using Guppy v5.0.7 (4). For the following stage, reads with read quality scores of ≥30 and 100-bp read lengths for Illumina sequencing and scores of ≥9 and 1,000-bp read lengths for ONT sequencing were used. After adapter trimming and quality checks with either Skewer v0.2.2 (5) or Porechop v0.2.4 (https://github.com/rrwick/Porechop), short reads (*n* = 8,984,838 reads [SalSpp05] or *n* = 5,869,998 reads [SalSpp10]) and long reads (*n* = 31,732 reads [average read length, 6,986.6 bases] [SalSpp05] or *n* = 34,848 reads [average read length, 6,355.2 bases] [SalSpp10]) were assembled using Unicycler v0.4.8 (6). The assembled genome was then checked for quality using QUAST v5.0.2 (7), and MOB-suite v3.0.3 was used for plasmid typing (8). Overall, the complete circular genomes of SalSpp05 (4,977,168 bp) and SalSpp10 (4,854,412 bp) contain one chromosome and two plasmids. Genome annotation was performed with the NCBI Prokaryotic Genome Annotation Pipeline (PGAP) v4.11 (9). All genome and plasmid features are summarized in Table 1. The average nucleotide identity (ANI) values for both genomes were calculated with GTDB-Tk v1.5.1 (10), which identified the strains as S. enterica on the basis of ~98% ANI with the reference genome (GenBank accession number AE006468.2). AMRFinderPlus v3.10.40 (11) predicted AMR genes in the aminoglycoside [*aadA2* (99.6% identity) and *aac(3)-IId* (100% identity)], lincosamide [*lnu*(F) (100% identity)], and β-lactam (*bla*_TEM-1_ [100% identity]) classes in the plasmid of SalSpp10 (GenBank accession number CP104489).

**TABLE 1:** Genome features of two S. enterica strains isolated from chicken carcass rinse water samples

Genome feature	Data for strain:					
	SalSpp05			SalSpp10		
	Chromosome	Plasmid 1	Plasmid 2	Chromosome	Plasmid 1	Plasmid 2
Size (bp)	4,964,371	8,873	3,924	4,806,677	44,362	3,373
Sequencing depth (×)	44.72	207.39	100.84	163.30	431.75	250.24
*N*_50_ (bp)	4,964,371			4,806,677		
GC content (%)	52.23	53.15	47.66	52.21	43.70	55.10
No. of CDSs^*a*^	4,847	9	4	4,660	61	5
No. of rRNAs	22	0	0	21	0	0
No. of tRNAs	86	0	0	82	0	0
ANI (%)	98.59			98.20		
GenBank accession no.	CP104491	CP104492	CP104493	CP104488	CP104489	CP104490

a CDSs, coding sequences.

### Data availability.

All complete circular genome sequences have been deposited in GenBank under the BioProject accession number PRJNA879268. The raw reads have been deposited in the Sequence Read Archive (SRA) under the accession numbers SRR21539850 and SRR21539849 (Illumina) and SRR21539845 and SRR21539844 (ONT) for strains SalSpp05 and SalSpp10, respectively.
